# A resting box for outdoor sampling of adult *Anopheles arabiensis *in rice irrigation schemes of lower Moshi, northern Tanzania

**DOI:** 10.1186/1475-2875-8-82

**Published:** 2009-04-25

**Authors:** Eliningaya J Kweka, Beda J Mwang'onde, Epiphania Kimaro, Shandala Msangi, Charles P Massenga, Aneth M Mahande

**Affiliations:** 1 Tropical Pesticides Research Institute, Division of Livestock and Human Diseases Vector Control, P.O. Box 3024, Arusha, Tanzania; 2 Tropical Pesticides Research Institute, Mabogini Field Station, Moshi, Tanzania

## Abstract

**Background:**

Malaria vector sampling is the best method for understanding the vector dynamics and infectivity; thus, disease transmission seasonality can be established. There is a need to protecting humans involved in the sampling of disease vectors during surveillance or in control programmes. In this study, human landing catch, two cow odour baited resting boxes and an unbaited resting box were evaluated as vector sampling tools in an area with a high proportion of *Anopheles arabiensis*, as the major malaria vector.

**Methods:**

Three resting boxes were evaluated against human landing catch. Two were baited with cow odour, while the third was unbaited. The inner parts of the boxes were covered with black cloth materials. Experiments were arranged in latin-square design. Boxes were set in the evening and left undisturbed; mosquitoes were collected at 06:00 am the next morning, while human landing catch was done overnight.

**Results:**

A total of 9,558 *An. arabiensis *mosquitoes were collected. 17.5% (N = 1668) were collected in resting box baited with cow body odour, 42.5% (N = 4060) in resting box baited with cow urine, 15.1% (N = 1444) in unbaited resting box and 24.9% (N = 2386) were collected by human landing catch technique. In analysis, the house positions had no effect on the density of mosquitoes caught (DF = 3, F = 0.753, P = 0.387); the sampling technique had significant impact on the caught mosquitoes densities (DF = 3, F 37. 944, P < 0.001).

**Conclusion:**

Odour-baited resting boxes have shown the possibility of replacing the existing traditional method (human landing catch) for sampling malaria vectors in areas with a high proportion of *An. arabiensis *as malaria vectors. Further evaluations of fermented urine and longevity of the urine odour still need to be investigated.

## Background

Several trapping techniques have been deployed in sampling malaria vectors population in the world [1-3]. Human landing catch has been considered as the gold standard method in mosquito sampling for surveillances and control programmes to estimate the infectivity rates, species abundance and mosquitoes dynamics [4]. With the increase of ethical issues in using humans as subject in collection of disease vectors, alternative simple methods, such as odour-baited traps, need to be developed and evaluated for their effectiveness [5]. Host odours provide olfactory cues by which haematophagous insects locate host for their blood meal [6]. Mosquitoes are the most important disease vectors in sub-Saharan Africa [7]. Odour-baited traps have shown to be effective to different disease vectors in tropical areas, including tsetse flies [8,9], mosquitoes [10] and ticks [11]. Several studies have shown the attractiveness of malaria vectors to different hosts [12,13]. In the *Anopheles gambiae *complex, studies have shown different odour preference among the sibling species: *Anopheles gambiae *s.s. is attracted by semiochemicals from humans sweat [14], *Anopheles arabiensis *notably varies from being attracted by humans or bovine odour, depending on the geographical location [15]. In several arid parts of sub-Saharan Africa, *An. arabiensis*, is a major malaria vector [16]. *Anopheles arabiensis *is regarded as more exophilic than other sibling species of the *An. gambiae *complex [15]. Several trapping tools, baited with odour, have shown great efficiency when evaluated for mosquitoes sampling in different areas, such as the mosquito magnet^® ^[17] and the MMX-trap [10]. In rural areas, there is a need to develop a trapping system, which uses the host-seeking strategies of the vectors and can be incorporated in simple tools/devices for vectors sampling [18,19]. The exploitation of both ecology and behavioural aspects of vectors are important in reducing the vector-human contact by developing targets or odour-based sampling tool [8,19].

The purpose of this paper was to report the findings of evaluating resting boxes baited with cow odour against human landing catch in irrigation schemes of lower Moshi, northern Tanzania.

## Methods

### Study area

The study was conducted at Mabogini village (37°20' E, 3°21'S and 800 M above sea level) in lower Moshi irrigation scheme area, northern Tanzania, as described elsewhere [13]. In this area, cattle owners do not share the shelter with livestock. Humans sleep in a house, while livestock and chickens have their separate constructed shelters (cowsheds). The village has a population of 20,614 with 4,871 households and an average of 4.2 people per house [19].

### Mosquitoes sampling

**Figure 1:**
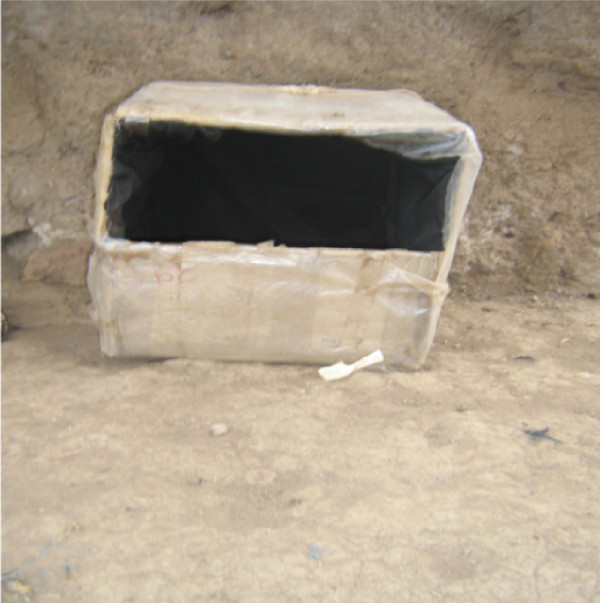
**Photograph showing the position of the box trap outdoor for *An. arabiensis *sampling**.

The four sampling techniques deployed were: human landing catch (HLC), box baited with cow body odour (BBCO), box baited with urine (BBU) and unbaited box (UB). These techniques were rotated between selected houses to avoid positional effect and bias. The boxes entry point was set at 30 centimeters above the ground outdoors (Figure 1). Boxes dimensions were 45 cm by 30 cm by 45 cm. These boxes where positioned horizontally to the surface such that mosquito entry point was on lateral side of the box. Collected female mosquitoes were graded according to abdominal conditions in each sampling technique. A total of 4 experiments were done per day in four houses (i.e. each sampling technique per house) for 30 days.

#### Box baited with cow body odour (BBCO)

The black wet cotton cloth was rapped on the body of insecticide-free cow under shade for one hour on experimental day. The cloth was folded in a plastic bag in freezer to keep the odour strong up to the experiment set up time.

#### Box baited with urine (BBU)

Black cotton clothing material was socked in fresh cow urine in the morning. It was then kept in a plastic bag in a freezer till experiment time after been dried to reduce wetness. Some fresh cattle urine was collected by a person in a cowshed from a urinating adult female cattle. The clothing material was used to cover the inner surface of the boxes during experimental set up.

#### Unbaited box (UB)

The black cotton cloth material used in this box was just of similar size as treated but had no any odour.

#### Human landing catch (HLC)

Collection of mosquitoes was performed by consenting personnel, as suggested by World Health Organization [4]. All experiments started at 18:00 hrs to 06:00 hrs.

### Statistical analysis

Data entry and validation was done in ms-excel 2003 version. Data analysis was performed using the SPSS version 15.0 for windows. General linear model was deployed to analyse the effect of all sampling tools and other factors such as days, relative to number of *An. arabiensis *collected in each house. The daily densities variation of mosquito caught in odour-baited resting and unbaited resting boxes were analysed using non-parametric or Kruskal-Wallis test, as data were not normally distributed. The abdominal conditions of the sampled mosquitoes were presented in percentages for each technique used.

### Ethical consideration

The permit of using of human as a subject was granted by KCM college of Tumaini university research ethics committee while TPRI proposal review team and institutional review board committee approved the other part of the study.

## Results

**Figure 2:**
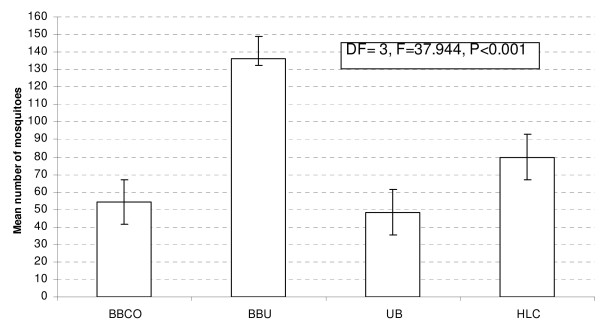
**Mosquito densities variation within techniques used in sampling malaria vectors in 30 days (The mean density was taken as the number of mosquitoes collected by each technique per 30 days)**. BBCO- Box baited with cow body odour (BBCO), Box baited with urine (BBU), Unbaited box (UB) and Human landing catch (HLC).

A total number of 9,558 of *Anopheles gambiae s.l *were collected. Of these 42.5% (n = 4060) by BBU, 24.9% (n = 2386) by HLC, 17.5% (n = 1668) were collected by BBCO, and 15.1% (n = 1444) by UB. All *An. gambiae s.l *collected in this area were regarded as *An. arabiensis *[13]. HLC did better in mosquitoes sampling than UB and BBCO (Figure 2).

In abdominal conditions, the percentage of unfed collected were 48.3%, 97.0%, 41.7% and 4.8%, fed were 0.0%, 3.0%, 16.2% and 4.6% semi-gravid were 22.7%, 0.0%, 0.0% and 92.0%, gravid were 29.0%, 0.0%, 42.1% and 0.6% for BBU, HLC, BBCO and UB respectively.

In evaluation of these resting boxes, a total of 128 (32 days) experiments were done. In analysis two days (eight experiments) were not considered for analysis due to delay in mosquito collection in the morning (i.e. mosquitoes were collected at 07:30 am instead of 06:00 am). General linear model univariate analysis method was used, position of the houses had no effect on mosquito density catch (DF = 3, F = 0.753, P = 0.387); the sampling techniques had significant impact on the caught mosquito densities as shown in Figure 2. The effect of days was not significant in the model (DF = 29, F = 5.095, P = 0.08). Mosquito density fluctuation between sampling tools found to be significant (X^2 ^= 50.806, DF = 29, P = 0.007) by the Kruskal-Wallis test.

## Discussion

The results of this study have demonstrated the usefulness of using simple tools for sampling disease vectors in surveillance and control programmes in disease endemic areas. These findings have been supported by previous studies of using different tools as observed in other studies [20-22]. BBU performed better than traditional method of human landing catch, the mostly known sampling method believed to perform well in malaria vector sampling in all positions [4]. The use of BBU in surveillance studies will give better results in areas with a higher population of *An. arabiensis *than HLC, hence protecting human from been bitten by infected mosquitoes during sampling exercise. These results have given an insight in proposing the need of re-evaluating the mosquito sampling method according to ecological factors or species abundance if already known. These boxes were able to collect mosquitoes outdoor without interfering with norms and values of the community that made the method to be more useful in the community. These boxes are freely obtained and affordable for surveillance studies in rural areas where modern trapping systems are limited. The complex natural animal odours used are available and not costly.

**Figure 3:**
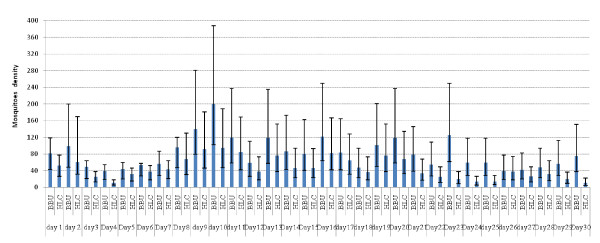
**The comparative efficiency of Box baited with urine (BBU) and Human landing catch (HLC) in mosquito collection daily for 30 days**.

The sampling of mosquitoes was not affected by other factor except trapping technique (Figure 2). This shows that in area with *An. arabiensis*, animal odours can be used to replace human landing catch technique, which was not powerful enough (Figure 3). Other methods of mosquito sampling, such as the pyrethrum spray catch (PSC) which samples indoor resting mosquitoes, needs well trained personnel, expensive instruments and entering peoples house fortnightly [4]. Light traps (LT) that sample host-seeking mosquitoes need electrical power to operate the traps that might not be available. These PSC and LT are performed indoors while *An. arabiensis *mosquitoes are more exophilic, exophagic and zoophilic [15]; hence PSC and LT might not perform well in areas with a high proportion of *An. arabiensis*. This study shows that cattle urine odour baited resting boxes had better results than traditional method (HLC) in collection of mosquitoes per day for 30 days (Figure 3).

This comparative study of HLC and odour-baited resting boxes enabled direct comparison of collection methods for *An. arabiensis *alternative to HLC. The main advantage of the odour-baited resting boxes is that it samples host-seeking mosquitoes and protects human from the risk of being bitten by infected mosquitoes [23]. The preference of cattle odour by *An. arabiensis *has been documented elsewhere [24]. It has also been recorded that the odour-baited traps promotes species-specific responses [13], which was further documented in this study (Figures 2 and 3).

This study showed similarity in attraction between BBCO and BBU in the proportions of unfed mosquitoes, which means that odour from any parts of the body can play the same role in surveillance studies for collection of *An. arabiensis *(Figure 2). In these sampling techniques, the high proportion of unfed mosquitoes was found in HLC, fed in BBCO, semigravid in UB and gravid in BBCO. This trend still convincing that, these techniques created specific abdominal status attraction [13]. The attraction of BBU to high mosquito density in different abdominal conditions should be observed critically to show the components responsible for such attraction, looking for example at what happens when urine ages. This method may be incorporated in surveillance studies for identifying the infectivity rates of malaria vectors and planning control measures for reducing malaria transmission in disease endemic areas [25].

## Conclusion

The use of complex natural odour baited resting boxes should be considered for further evaluation This will be more appropriate in planning for the intervention or control measures against *An. arabiensis*. The ecological characteristics should be used to deploy the appropriate odour for vector sampling.

## Competing interests

The authors declare that they have no competing interests.

## Authors' contributions

EJK conceived and designed the study, data collection and analysis; and writing up of the first draft of this manuscript. BJM and CPM involved in data collection. SM and EEK provided support on the overall study and commented on drafts of this manuscript. AMM co-designed and was responsible for data collection and analysis. All authors read and approved the final version of this manuscript.
